# Stability modification of therapeutic aptamers: from biostability bottlenecks to nuclease-resistant construct design

**DOI:** 10.1039/d6cb00062b

**Published:** 2026-05-06

**Authors:** Zhuoheng Pan, Yufei Pan, Huarui Zhang, Zefeng Chen, Yihao Zhang, Baoting Zhang, Aiping Lu, Ge Zhang, Sifan Yu

**Affiliations:** a Law Sau Fai Institute for Advancing Translational Medicine in Bone and Joint Diseases (TMBJ), School of Chinese Medicine, Hong Kong Baptist University Hong Kong SAR China aipinglu@hkbu.edu.hk zhangge@hkbu.edu.hk yusifan@hkbu.edu.hk; b Aptacure Therapeutics Limited Hong Kong SAR China; c School of Chinese Medicine, Faculty of Medicine, The Chinese University of Hong Kong Hong Kong SAR China; d Shenzhen Institute for Research and Continuing Education (IRACE), Hong Kong Baptist University Shenzhen China

## Abstract

Nucleic acid aptamers are synthetic, single-stranded oligonucleotides that bind targets with high affinity and specificity, enabling precise and programmable functional modulation. However, low biostability remains a key druggability bottleneck of therapeutic aptamers. *In vivo* enzymatic lability constitutes a primary pharmacokinetic barrier, impeding systemic exposure, tissue distribution, and sustained target occupancy, all critical determinants of *in vivo* efficacy. Consequently, rational chemical stabilization *via* enzyme-resistant modifications has emerged as a cornerstone strategy in aptamer drug development. In this review, we systematically categorize current stabilization approaches across two complementary design dimensions: local-level modifications (including terminal modification, sugar modification, backbone modification, and base modification) and global structural-level engineering (including Spiegelmers, cyclization modification, and multivalent assembly). Furthermore, we discuss persistent translational challenges to illuminate a coherent framework for designing aptamers with high biostability that achieve enhanced nuclease resistance while concurrently exhibiting favorable pharmacokinetics, high target binding affinity and specificity.

## Abstract 2

Nucleic acid aptamers are synthetic, single-stranded oligonucleotides that bind targets with high affinity and specificity, enabling precise and programmable functional modulation.

## Graphical abstract

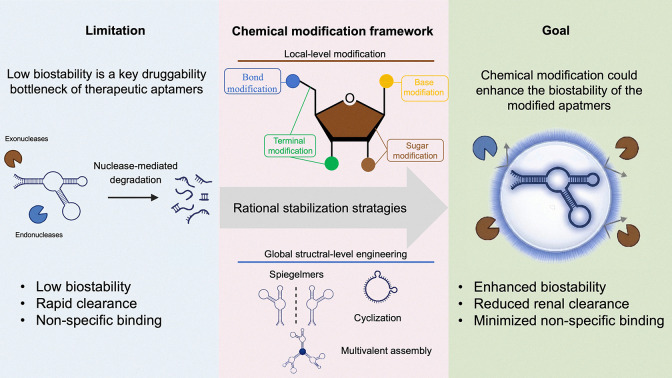

## 1. Introduction

Aptamers are synthetic, single-stranded DNA or RNA oligonucleotides that fold into defined three-dimensional conformations capable of binding molecular targets—most commonly proteins—with high affinity and specificity. In contrast to antisense oligonucleotides or siRNAs, which rely primarily on sequence-complementary base pairing, aptamers function through structure-dependent molecular recognition: tertiary motifs such as hairpins, internal loops, junctions, pseudoknots, and G-quadruplexes cooperate to generate shaped binding surfaces that can discriminate subtle physicochemical features on a target.^1,2^ Because they can approximate antibody-like target engagement while remaining fully chemically programmable and manufacturable by solid-phase synthesis, aptamers are frequently described as “chemical antibodies.”^1^

The predominant methodology for aptamer discovery is systematic evolution of ligands by exponential enrichment (SELEX), in which iterative cycles of binding, partitioning, and amplification enrich rare target-binding sequences from highly diverse random libraries.^3,4^ Since its introduction, SELEX has matured into an increasingly standardized workflow, benefiting from improvements in selection design (*e.g.*, more stringent counter-selection), analytical readouts that accelerate enrichment tracking, and automation-friendly formats. These developments have expanded the accessible target space and facilitated the generation of aptamers against a wide range of soluble proteins, receptors, and other biomolecular targets relevant to therapeutic intervention.^5^

From a drug-development perspective, aptamers occupy a distinctive niche between small molecules and biologics. Similar to small molecules, they can be produced by scalable chemical synthesis with strong batch-to-batch consistency and precise control over composition. Their nucleic-acid scaffold supports position-defined, modular chemical engineering, enabling the installation of functional groups, linkers, imaging labels, or drug payloads at predetermined sites.^6^ At the same time, like monoclonal antibodies, aptamers can achieve nanomolar, even picomolar binding affinities and exceptional selectivity through conformationally encoded recognition surfaces.^7,8^ In principle, this combination of programmability, manufacturability, and high-performance binding makes aptamers attractive as ligands for extracellular blockade, receptor modulation, targeted delivery, and multi-specific therapeutic assemblies.

Despite these conceptual advantages, the therapeutic translation of aptamers has been repeatedly constrained by intrinsic developability bottlenecks that limit durable *in vivo* activity. Among the most frequently cited challenges are: (i) limited biostability due to nuclease-mediated degradation in biological fluids and tissues; (ii) rapid clearance—particularly renal filtration—driven by low molecular weight and high hydrophilicity; (iii) non-specific interactions that reshape biodistribution and reduce free, target-accessible concentrations; and (iv) context-dependent immune stimulation or other safety liabilities associated with certain sequences, chemistries, or formulations.^1,9^ While the relative importance of these factors varies by indication, route of administration, and target biology, inadequate *in vivo* persistence is often the most immediate barrier, because loss of sequence integrity or disruption of the active fold rapidly abolishes target engagement and shortens the pharmacologically effective exposure window.^10^ Consequently, improving stability is not simply a downstream optimization; it is frequently a prerequisite for converting strong *in vitro* binding into durable *in vivo* pharmacology and clinically practical dosing.

Over the past two decades, numerous stability modification approaches have been developed to improve aptamer performance *in vivo*, ranging from chemical changes to higher-order construct engineering. However, these stability modifications of aptamers are rarely interchangeable: they can reshape folding, binding behavior, exposure, safety, and manufacturability, making the selection and combination of modification elements a key practical challenge. In this review, we recapitulate aptamer stability modification strategies with an emphasis on design logic and translational trade-offs. We first provide a concise overview of key developability challenges and explain how stability constraints shape dosing feasibility and the therapeutic window. We then survey major classes of aptamer stability modification, including terminal, sugar, backbone, and base-level chemistries, followed by architecture-level approaches such as cyclization and nuclease-orthogonal designs. Where relevant, we discuss how stability modification choices interact with exposure, biodistribution, and safety considerations, and we highlight practical decision points (*e.g.*, RNA *vs.* DNA scaffolds, anticipated nuclease environment, target residence-time requirements, and manufacturability constraints) that guide selection of a modification strategy. Our goal is to offer a structured and actionable framework that helps researchers move from “a high-affinity binder” to an aptamer construct with improved *in vivo* robustness, thereby enabling a clearer path toward therapeutic translation.

## 2. Low biostability is a key druggability bottleneck of therapeutic aptamers

Low biostability remains a key druggability bottleneck of therapeutic aptamers. Although aptamers can achieve high affinity and specificity *in vitro*, their function *in vivo* depends on maintaining both sequence integrity and a binding-competent tertiary fold under physiological conditions. Because aptamer recognition is conformation-driven, partial degradation that would be tolerable for some linear oligonucleotide modalities can be functionally catastrophic for aptamers: cleavage at a structurally critical loop, junction, or stabilizing stem can trigger global unfolding and rapid loss of target engagement.^11,12^ As a result, Low biostability often emerges as the earliest and most stringent barrier when translating an *in vitro*-selected binder into an *in vivo*-active therapeutic construct.

### 2.1. Nuclease-mediated degradation pathways

The dominant mechanistic driver of poor aptamer biostability is nuclease-mediated degradation in biological fluids and tissues. Endonucleases cleave internal phosphodiester linkages, whereas exonucleases progressively trim oligonucleotides from the 3′ and/or 5′ termini.^13^ These processes occur concurrently *in vivo*, and the truncated fragments produced of the required structural motifs may not be able to maintain the high-affinity recognition of the aptamer.^14^ Importantly, nuclease attack does not need to be extensive to abolish function: a single internal cut or a modest degree of terminal trimming can disrupt long-range tertiary contacts and destabilize the active fold.

RNA aptamers are generally more vulnerable than DNA aptamers. Beyond abundant RNase activity, the presence of the 2′-hydroxyl group increases intrinsic backbone lability and can facilitate cleavage reactions that accelerate degradation. DNA aptamers lack the 2′-OH and therefore tend to exhibit improved intrinsic stability, yet they remain susceptible to DNases and can still undergo rapid degradation depending on sequence context and fold topology. Consequently, Low biostability is a pervasive constraint across aptamer scaffolds, with severity determined by both chemical composition (RNA *vs.* DNA) and structural features that create nuclease-accessible sites.^10,15^

### 2.2. Structure–stability coupling and loss of biological function

A defining feature of aptamers is the tight coupling between structure and function: affinity and specificity arise from a precise three-dimensional arrangement of nucleotides. This coupling makes aptamers especially sensitive to degradation. Terminal truncation can remove base-pairing elements that stabilize stems and coaxial stacking; internal cleavage can collapse loop architectures and multi-helix junctions that organize the binding interface; and local destabilization can propagate to global conformational rearrangement.^16^ Consequently, the functional impact of degradation could be nonlinear—particularly for structurally sensitive aptamers (*e.g.*, those adopting stem-loop conformations), where even minor chemical damage can produce unpredictable losses in binding affinity, altered binding kinetics, or reduced specificity.^17–19^ This structure–stability relationship explains why improving nuclease resistance is typically a prerequisite for sustaining aptamer activity in complex biological matrices and for enabling therapeutically meaningful target engagement.

### 2.3. Translational implication: why stability modification is embedded in advanced constructs

Because unmodified aptamers are prone to rapid nuclease degradation, stability modification is commonly integrated early in therapeutic design rather than treated as a late-stage optimization. In practice, aptamers that advance toward late preclinical development or clinical evaluation are rarely “naked” oligonucleotides; instead, they are engineered constructs incorporating stability modification elements to preserve the active fold and maintain target affinity *in vivo*. A canonical example is pegaptanib, the first FDA-approved aptamer therapeutic, whose drug-like behavior depends on an integrated modification stack rather than the native sequence alone.^20^ More broadly, real-world translational designs frequently employ multiple, complementary stability modifications, reflecting a practical principle: robust nuclease resistance typically requires combinatorial protection against both exo- and endonuclease pathways while maintaining structural determinants of binding. To connect these translational examples to the mechanistic basis outlined above, Table 1 summarizes approved and representative clinical-stage aptamer therapeutics together with the principal stability modification stacks used in each construct.

**Table 1:** Marketed and representative clinical aptamers, with modifications used

Aptamer	Target	Indication	Status	Route/format	Modifications used for enhancing biostability	Ref.
Pegaptanib (Macugen)	VEGF165	Neovascular (wet) AMD	Approved	Intravitreal; PEGylated RNA aptamer	2′-fluoro pyrimidines; 2′-*O*-methyl purines; 3′ inverted dT cap; 5′ PEG (∼40 kDa)	20
Avacincaptad pegol (Izervay)	Complement C5	Geographic atrophy (AMD)	Approved	Intravitreal; PEGylated RNA aptamer	Branched PEG (∼43 kDa); reported fluorinated and *O*-methylated ribose substitutions	21
Pegpleranib (Fovista/E10030)	PDGF-B pathway	Wet AMD (combo trials)	Phase 3 completed; not approved	Intravitreal; PEGylated aptamer	PEGylation; 2′-fluoro pyrimidines; 2′-*O*-methyl purines	22
Rondaptivon pegol (BT200)	vWF A1 domain	Type 2B VWD/hemophilia A (FVIII extension)	Clinical (multiple trials)	Subcutaneous; PEGylated RNA aptamer	Fully 2′-*O*-methylated RNA aptamer; PEG (∼40 kDa)	23
ARC1779	vWF A1 domain	Antithrombotic/aTTP (historical trials)	Clinical (historical)	PEGylated DNA/RNA hybrid	2′-*O*-methyl nucleotides; phosphorothioate linkages; 3′ inverted dT; 5′ PEG (∼20 kDa)	24
Pegnivacogin (RB006; REG1 system)	Factor IXa	ACS/PCI anticoagulation (with antidote)	Clinical program terminated (historical)	IV; PEGylated RNA aptamer + complementary antidote	5′ PEG (∼40 kDa) conjugation; paired antidote oligo	25
Olaptesed pegol (NOX-A12; Spiegelmer)	CXCL12 (SDF-1)	Oncology/immuno-oncology combinations (representative)	Phase 1/2 reported	PEGylated l-oligonucleotide	l-stereoisomer oligonucleotide; PEG (∼40 kDa)	26
Lexaptepid pegol (NOX-H94; Spiegelmer)	Hepcidin	Anemia of chronic disease (representative)	Phase 2 reported	PEGylated l-oligonucleotide	l-stereoisomer oligonucleotide; PEG (∼40 kDa)	27
BAX499 (ARC19499)	TFPI	Hemophilia (historical)	Clinical program terminated (historical)	PEGylated modified RNA aptamer	PEG conjugation; 2′-*O*-methyl purine modifications reported; 3′ inverted dT cap reported	28

Building on this mechanistic understanding, the next section systematically reviews major classes of aptamer stability modification strategies and discusses how they can be combined into compatible stacks that preserve folding and binding function.

## 3. Stability modification strategies for enhancing the nuclease resistance of aptamers

Building on the nuclease-driven mechanisms of limited aptamer biostability discussed in Section 2, this section summarizes aptamer stability modification strategies that enhance resistance to degradation while preserving the binding-competent fold. We focus on two complementary design layers: (i) intrinsic chemical modifications and terminal protection that directly reduce nuclease susceptibility, and (ii) construct-level engineering—such as end-conjugation, cyclization, chirality inversion, and multivalent assembly—that improves stability mainly through steric shielding and conformational constraint. Because these interventions can also influence folding, binding kinetics, and developability, the practical aim is to assemble a compatible modification stack that maximizes functional persistence in biological environments without compromising target recognition.

### 3.1. Terminal stability modification and end-conjugation

Terminal stability modification primarily protects aptamers from exonuclease-mediated trimming by masking free 3′/5′ ends, either *via* small end-caps or *via* end-conjugation that creates steric hindrance at the termini.

#### 3.1.1. Biotin modification

Modifying the 3′ end with biotin (vitamin B7) is a widely used strategy. Under this strategy, biotin provides steric protection against the nuclease activity of aptamers and facilitates the affinity purification and detection of aptamers. The main mechanism by which 3′-biotin confers stability is the steric hindrance effect. Serum 3′-exonucleases such as snake venom phosphodiesterase need to form a specific stereochemical match with the free 3′-hydroxyl group to initiate the hydrolysis reaction. The biotin group linked by a flexible linker such as triethylene glycol will disrupt this enzyme-substrate interface.

The protective effect of 3′-biotin is significant in *in vitro* experiments. Shum *et al.* developed a non-*G*-quadruplex DNA aptamer (NG8) targeting the SARS coronavirus helicase and conducted research on its 3′-biotin modification. The results showed that 3′-biotin modification can enhance its stability against 3′-exonuclease and maintain the specificity and helicase inhibitory activity of the aptamer before modification. Compared with the unmodified variant, 3′-biotin modification enabled NG8 to remain stable for 31 and 16 hours in 5% and 10% fetal bovine serum respectively, while the unmodified aptamer only lasted for 16 and 6 hours, doubling the duration. Streptavidin is used to enhance the steric hindrance effect of 3′-biotin-modified aptamers due to its non-covalent interaction with biotin, which has extremely high affinity and stability. Meanwhile, the increase in molecular weight can also effectively reduce the renal clearance rate.^29^ Dougan *et al.* developed 3′-biotin and 3′-biotin–streptavidin modification methods for two DNA aptamers targeting human α-thrombin respectively and verified them *in vitro* and in different animal models (mice and rabbits). 3′-biotin–streptavidin not only showed good resistance to blood nucleases *in vitro* but also greatly reduced their degradation efficiency *in vivo*. Compared with the aptamers without biotin–streptavidin modification, the degradation efficiency was reduced by 10 to 20 times. At the same time, their affinity for thrombin was retained.^30^

#### 3.1.2. 3′–3′ linkage modification

The 3′–3′ linkage can also be referred to as inverted deoxyThymidine (idT) modification. IdT modification involves adding an inverted thymidine cap to the 3′ end of the oligonucleotide, which prevents the exposure of the 3′-OH group. Thus, it protects the aptamer terminus from being digested by exonucleases. IdT is the most common modification currently, and this modification is frequently used in the marketed aptamer drug Macugen and aptamer candidate drugs in the clinical stage.^31^ Therefore, idT modification is indispensable in the transformation of aptamer drugs. This modification method was first reported by Shaw *et al.* and was applied to the modification of an 11-*mer* oligonucleotide sequence, which significantly improved its stability in serum.^32^ This method uses a modified controlled pore glass (CPG) carrier. The 5′-hydroxyl group of the initial 3′-end nucleoside is connected to the solid carrier. The next nucleoside phosphoramidite will be coupled with the exposed 3′-hydroxyl group, initiating the classic 3′ → 5′ chain extension reaction, and terminating the “tail” of the aptamer in a 3′–3′ linkage manner. Ortigao *et al.* subsequently extended this 3′-inverted nucleotide strategy to antisense oligonucleotides (ASO), demonstrating improved stability in human serum.^33^ Consistently, Dass *et al.* applied idT capping to a 33-*mer* DNAzyme and observed a marked enhancement in serum stability, with the half-life increasing from 70 min to 22 h compared with the unmodified construct. It also enhanced the ability of the DNAzyme to enter cells.^34^ When compared with the aptamer NG8 with a single 3′-biotin modification, the NG8 with a single idT modification showed stronger resistance against exonucleases.^30^

#### 3.1.3. Modified nucleoside capping modification

**Fig. 1:**
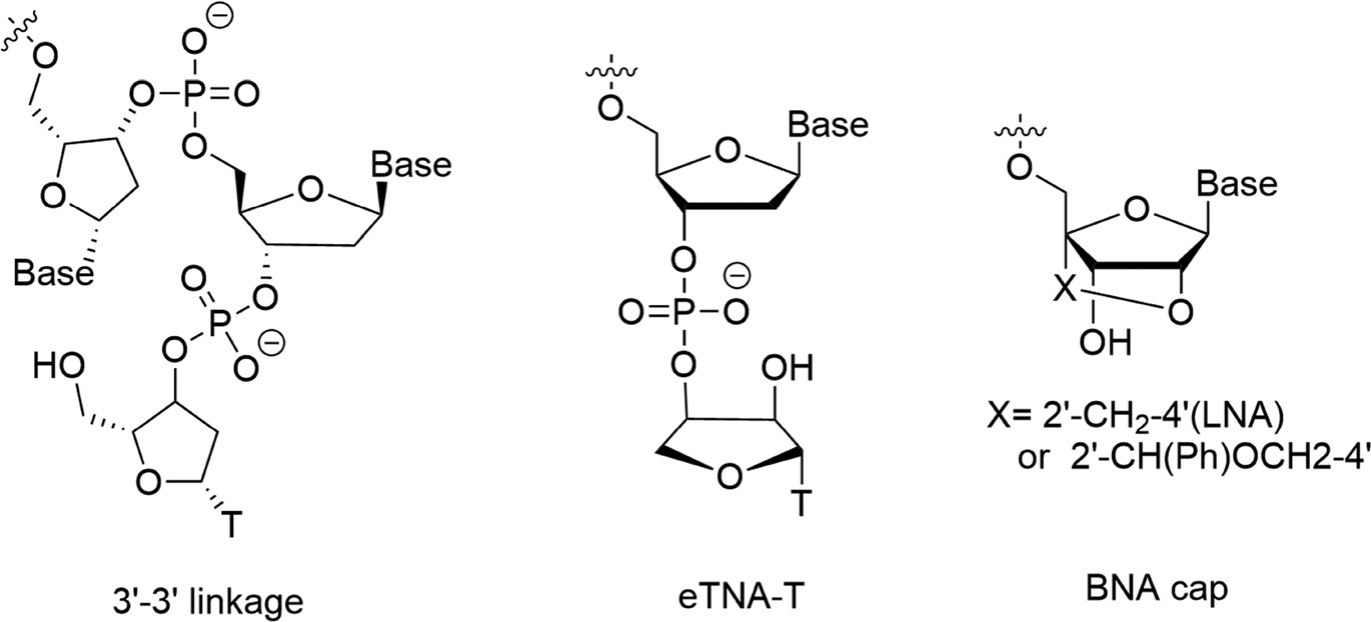
Modified nucleoside capping modification, including 3′–3′ linkage, Etna, BNA cap.

Modified nucleosides are usually incorporated into oligonucleotide sequences through chemical synthesis before SELEX to confer resistance to endonuclease cleavage. However, recently, post-SELEX modification of the 3′ end has emerged as a powerful strategy to specifically prevent 3′ → 5′ exonuclease degradation (Fig. 1). Kasahara *et al.* systematically evaluated the effects of several bridged nucleotide (BNA) analogues, including locked nucleic acid (LNA) and phenyl-substituted BNA with a 2′-CH(Ph)OCH_2_–4′ bridge, on the enzymatic stability of the thrombin-binding aptamer TBA1. In the powerful 3′ exonuclease snake venom phosphodiesterase (VPD), LNA capping increased the stability of TBA1 by 3.6 times compared to the unmodified control; this improvement was also confirmed in human serum, where stability increased by 1.5 times. The phenyl-substituted BNA capping provided even stronger protection, with stability in VPD and human serum increasing by 27 times and 3.3 times, respectively, compared to the unmodified TBA1. This enhanced protection may be due to the steric hindrance caused by the larger volume of the phenyl group, but this increased stereochemical hindrance also affected the enzymatic ligation by terminal deoxynucleotidyl transferase (TdT), requiring higher synthetic demands. Notably, the introduction of these BNA modifications did not affect the binding affinity of the aptamer to its target.^35^ Recently, Wen *et al.* reported a novel modified nucleoside, eTNA, which combines the structural features of inverted dT and TNA. In eTNA, the 3′-oxygen is directly connected to the phosphate backbone, while the sugar configuration is reversed, forming a conformationally restricted, reverse-oriented thymidine cap. When applied to DNA aptamers, eTNA capping provided exceptional biostability: no degradation was detected in VPD within 72 hours, while the unmodified aptamer was completely degraded within minutes; in 50% human serum, the half-life of the aptamer was extended from 27.6 minutes to 24.2 hours. Importantly, under the same conditions, eTNA outperformed idT, with a 2.3-fold increase in stability - indicating its superiority as a next-generation 3′-protecting group.^36^

#### 3.1.4. Pegylation (PEG) modification

PEG modification is generally regarded as a common strategy to limit the renal filtration rate of oligonucleotides. Since it significantly increases the molecular weight of oligonucleotide molecules, this modification usually occurs at the 5′-end. In terms of serum stability, PEG is also considered to have a protective effect on aptamers against exonucleases. High-molecular-weight PEG chains create a hydrated, sterically crowded physical barrier that thermodynamically excludes nucleases from accessing the susceptible phosphodiester backbone, thus resisting exonuclease degradation from the 5′-end.^37,38^ Hoffmann *et al.* were the first to couple a single linear PEG to an amino-modified, chirally inverted RNA aptamer NOX-E36 and verified its better nuclease resistance.^39^ Haruta *et al.* developed a new double-branched pegylation method called symmetrical branching 2-cyanoethyl-*N*,*N*-diisopropyl phosphoramidite PEGylation (sbC-PEGylation). This method was applied to the 5′ ends of the RNA aptamers 17M-382 and 17M-200-S1 targeting the IL-17A inhibitor. Pharmacokinetic studies in mice showed that compared with the unmodified aptamers and those only modified with double-branched PEG coupling, the sbC-PEGylated polymers exhibited better stability in the blood circulation of both mice and monkeys. This may be due to the more significant steric hindrance induced by the introduced sbC fragment, which inhibits nuclease degradation.^40^ Recently, Zhang's team reported a poly serinol phosphodiester (PSP) polymer-assisted compaction (pac) Aptamer containing highly branched PEG modification. It is a brush-type polymer with a PEG chain as the backbone. The pharmacokinetics of the DNA aptamer HD1 modified with PSP pac in mice showed that the duration of PSP pac HD1 in the blood was significantly longer than that of free HD1. The difference in the total *in vivo* drug exposure (AUC_0_ → ∞) was 16-fold, and it avoided the immunogenicity caused by traditional linear PEG modification. This modification method improves the performance of the aptamer drug while ensuring its safety.^41^

#### 3.1.5. Lipid modification

Cholesterol conjugation is one of the most widely studied lipid-based aptamer stability modifications. Its primary value lies in improving nuclease resistance: the cholesterol moiety associates with circulating lipoproteins (*e.g.*, LDL and HDL), creating steric shielding that reduces nuclease access—particularly at susceptible termini—and thereby slows exonuclease-driven trimming. As a practical consequence, enhanced nuclease protection is often reflected in improved persistence in biological matrices and, in many cases, a prolonged circulation half-life *in vivo*.^42–44^ Smidt *et al.* first demonstrated that cholesterol-modified oligonucleotides anchor to the surfaces of LDL and HDL isolated from human plasma. Consistent with a stability-driven benefit, cholesterol conjugation led to an ∼10-fold increase in plasma half-life in rats compared with the unconjugated form, and the modified oligonucleotides also exhibited delayed degradation in rat serum.^45^ Aliyu *et al.* further implemented a cholesterol strategy using a triethylene glycol (TEG) linker (COL-TEG), attaching it to the 3′ end of the DNA aptamer OKA_24 and the 5′ end of OKA_26 (heme-targeting). Both cholesterol-TEG conjugates displayed improved nuclease resistance in serum while maintaining, and in their system improving, binding performance.^46^

Beyond cholesterol, diacylglycerol (DAG) has also been explored as a lipid-based stability modification, but it typically requires an exogenous lipid stabilization system. In this design, DAG serves as a membrane anchor that inserts the aptamer into liposomal bilayers, which physically shields the oligonucleotide from nuclease attack. Willis *et al.* applied this approach to the VEGF-binding RNA aptamer NX213 by incorporating DAG-NX213 into liposomes *via* sonication. Under RNase T1 challenge, approximately one-third of the liposome-associated DAG-NX213 remained protected, whereas free DAG-NX213 was completely cleaved. *In vivo*, this nuclease shielding translated into improved persistence: DAG-NX213 showed a longer half-life than the naked NX213, and liposomal incorporation further reduced plasma clearance relative to the free DAG-NX213.^47^

#### 3.1.6. Galactosylated modification

The strategy of glycosylated modified oligonucleotides represents the current frontier research in active tissue-targeted therapy. Among them, oligonucleotide drugs such as siRNA, ASO, and aptamer conjugated with trivalent *N*-acetylgalactosamine (Tri-GalNAc) structures have been proven to specifically target the asialoglycoprotein receptor (ASGP-R) on hepatocytes with high affinity. This targeted glycosylation modification has been applied in marketed drugs. Meanwhile, in these successful drug deliveries, it has also been found that the introduction of carbohydrates can improve the stability of oligonucleotides against nucleases.^48^

Tan's team developed a novel glycosylation strategy, designed and synthesized four glyconucleic acid modules. Through the Sonogashira cross-coupling reaction, the glycoside moiety was connected to the uracil rings at different positions of the aptamer, resulting in the formation of glyconucleic acid aptamer(GNAA). This strategy was applied to the DNA aptamer Sgc8 targeting the PTK7 protein, and their stability was evaluated both *in vivo* and *in vitro*. The results showed that glycosylation modification at the 3′ and 5′ ends could significantly improve the serum stability of the DNA aptamer while maintaining its structure and high affinity. Among them, the terminal modification of GalNAc exhibited 14-fold higher stability compared to the unmodified Sgc8, showing the best performance among the four modifications. The detection of metabolic effects and tumor-targeting ability in mice confirmed that its activity and safety were not interfered with by the glycosylation strategy. In terms of the mechanism of action, molecular dynamics simulation revealed the interaction mechanism between the Sgc aptamer with 3′ modification of GalNAc and exonuclease I (EXO1). Under this modification, the number of interactions between the bases of the Sgc aptamer and EXO1 residues decreased from 6 to 3 compared with that of the unmodified Sgc aptamer. The terminal glycosylation structure reduced the binding energy between the 3′ end of the aptamer and the active site of exonuclease I, decreased the binding affinity of the aptamer to the nuclease, and enhanced the aptamer's resistance to enzymatic degradation. As a simple, economical and efficient new strategy, GNAA has the potential to be introduced into more aptamers and oligonucleic acid drugs.^49^

### 3.2. Sugar ring modifications

Sugar ring modifications represent a critical approach in aptamer engineering to bolster their resistance to nucleases, enhance thermal stability, and improve overall pharmacokinetic properties for biomedical applications. These alterations target the ribose moiety, often conferring structural rigidity or flexibility that optimizes binding affinity and durability in biological environments.

#### 3.2.1. 2′-F, 2′-NH_2_, and 2′-OMe modifications

**Fig. 2:**
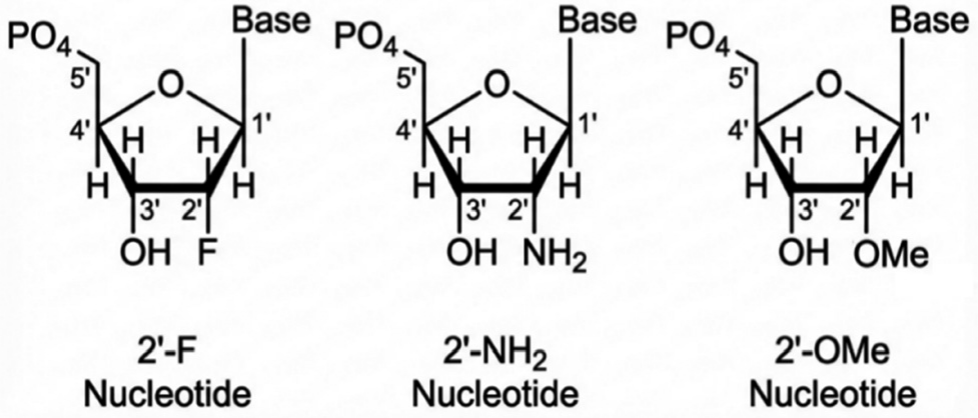
Ribose modifications, including 2′-fluoro (2′-F) modification, 2′-NH_2_ modification, 2′-OMe modification.

Modifications at the 2′ position of the ribose—most commonly 2′-fluoro (2′-F), 2′-amino (2′-NH_2_), and 2′-*O*-methyl (2′-OMe)—are among the most widely used aptamer stability modifications to enhance resistance to nuclease-mediated degradation (Fig. 2).^50–52^ Mechanistically, these substitutions replace the native 2′-hydroxyl group, a key structural feature recognized and exploited by many RNases, thereby reducing productive enzyme–substrate interactions and slowing backbone cleavage. As a practical benefit, improved nuclease resistance increases functional persistence in biological matrices and is often accompanied by longer apparent serum half-life.

Among these chemistries, 2′-F substitution is frequently used to strengthen nuclease resistance and can also stabilize certain structural motifs (including G-quadruplexes) when appropriately positioned. In reported thrombin aptamers, 2′-F incorporation has been associated with enhanced resistance to degradation in fetal bovine serum, with some cases also showing improved binding performance.^53,54^ Similarly, 2′-NH_2_ substitutions have been reported to markedly improve serum stability for aptamers against targets such as human neutrophil elastase and vascular endothelial growth factor, consistent with the role of 2′-chemistry in suppressing nuclease susceptibility.^55^ The 2′-OMe modification is particularly attractive for post-selection optimization because it can substantially improve nuclease resistance while maintaining binding performance, and it is commonly combined with 2′-F in clinically optimized designs (*e.g.*, anti-VEGF aptamers).^52^ Notably, different 2′ chemistries may differ in immunological profiles; for example, 2′-F has been reported to carry a higher risk of innate immune activation in certain contexts, whereas 2′-OMe is often considered more immunologically silent.

#### 3.2.2. 4′-thio modification

**Fig. 3:**
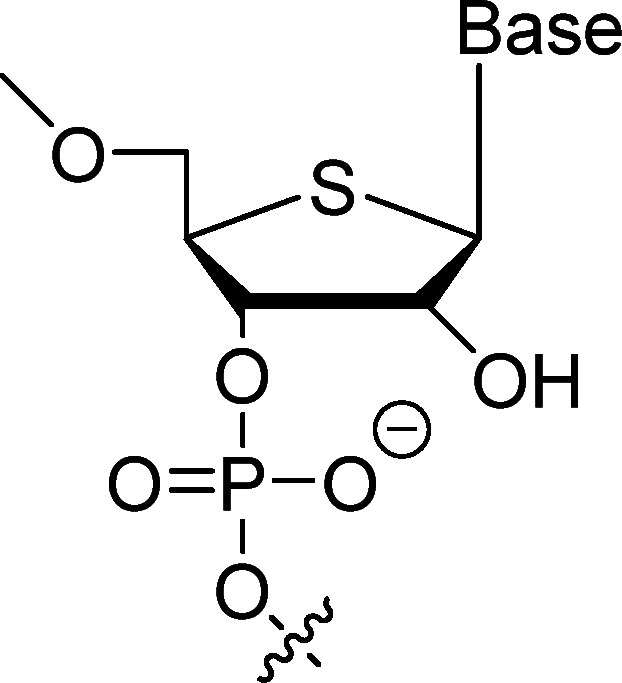
Structure of 4′-thio modification.

The 4′-thio modification involves replacing the 4′-oxygen in the sugar ring with sulfur, which significantly enhances resistance to RNases without compromising binding specificity (Fig. 3).^31^ This alteration has been applied in *in vitro* selection processes using 4′-thioUTP and 4′-thioCTP, yielding thioRNA aptamers with superior stability. A notable example is a 4′-thio-modified anti-thrombin aptamer that exhibits a 50-fold increase in RNase A resistance compared to unmodified RNA, alongside high-affinity binding (*K*_d_ = 4.7 nM).^56^ Optimized selections have further improved affinity to 7.2 nM, demonstrating the modification's utility in generating biostable aptamers for therapeutic targets like thrombin.

#### 3.2.3. Locked nucleic acid (LNA)

**Fig. 4:**
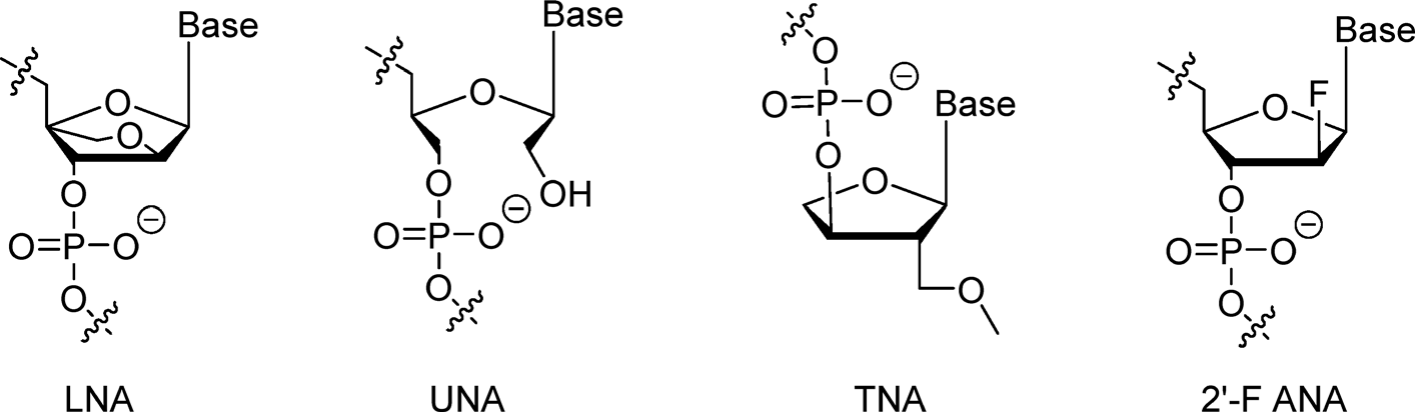
Structures of locked nucleic acid (LNA), unlocked nucleic acid (UNA), threose nucleic acid (TNA) and 2′-deoxy-2′-fluoro-d-arabinonucleic acid (2′-F ANA).

Locked nucleic acids (LNAs) introduce a methylene bridge between the 2′-oxygen and 4′-carbon atoms (Fig. 4), constraining the ribose in a 3′-endo conformation that elevates thermal stability and resistance to degradation.^57–59^ This rigid structure increases duplex melting temperatures and nuclease resistance, making LNA ideal for aptamer therapeutics.^60^ In selections using mutant T7 RNA polymerases, LNA-T and LNA-A incorporations, often paired with 2′-F pyrimidines, produced aptamers targeting influenza hemagglutinin and human CD40 ligand with low-nanomolar affinities and minimal degradation (<20% loss after 4 days in 25% human serum at 37 °C).^61^ However, high LNA content is sequence-dependent, and modifications in loops may contribute less to stability. LNA also risks hepatotoxicity in some contexts.

#### 3.2.4. Unlocked nucleic acid (UNA)

Unlocked nucleic acids (UNAs) disrupt the C2′–C3′ bond in the ribose ring (Fig. 4), introducing acyclic flexibility that facilitates conformational adjustments and promotes an induced-fit binding mechanism.^60^ This modification reduces duplex thermal stability but enhances aptamer adaptability, improving affinity for certain targets.^62^ In a 31-nucleotide anti-thrombin DNA aptamer (RE31), incorporating UNA-C at position T15 yielded a *K*_d_ value of 0.43 nM, a marked improvement over the unmodified 1.34 nM, with similar gains for UNA-A, UNA-U, and UNA-G variants (0.68–0.76 nM).^63^ UNA thus serves as a tool for fine-tuning stability and binding in G-quadruplex-containing aptamers, though it may destabilize triplex formations in some structures.

#### 3.2.5. Threose nucleic acid (TNA)

Threose nucleic acids (TNAs) feature a simplified four-carbon sugar (threose) where the phosphate backbone connects directly to the 3′ carbon (Fig. 4), conferring enhanced resistance to enzymatic digestion compared to DNA or RNA.^64^ This modification supports Watson–Crick base pairing while improving biostability, making TNA suitable for aptamer chimeras. Enzymatic resistance assays show TNA-based probes resist degradation that plagues DNA counterparts, avoiding false-positive signals in detection applications. TNA extensions at the 3′ terminus of DNA oligonucleotides *via* polymerase yield chimeras highly resistant to exonuclease I, with tGTP-extended products showing variable but improved durability.^65^ Additionally, TNA exhibits greater acid stability than RNA, with slower depurination rates under acidic conditions.

#### 3.2.6. Arabinonucleic acid (ANA) and 2′-F-ANA (FANA)

Arabinonucleic acids (ANA) and their 2′-fluoro derivative (FANA) are stereoisomers with the 2′ substituent in the “up” (arabino) configuration (Fig. 4), differing from the natural “down” (ribo) form.^66^ These modifications enhance nuclease resistance and thermal stability while enabling RNase H activation upon hybridization to RNA, which is valuable for gene silencing therapies. ANA/RNA hybrids serve as RNase H substrates, promoting target cleavage. FANA, in particular, hybridizes strongly to RNA, induces RNase H-mediated degradation, and shows high serum stability; phosphorothioate FANA is commonly used in antisense oligonucleotides.^67^ FANA aptamers targeting HIV-1 integrase and reverse transcriptase exhibit low-nanomolar affinities and conformational rigidity, with applications in inhibiting viral activity.

### 3.3. Phosphodiester bond modifications

**Fig. 5:**
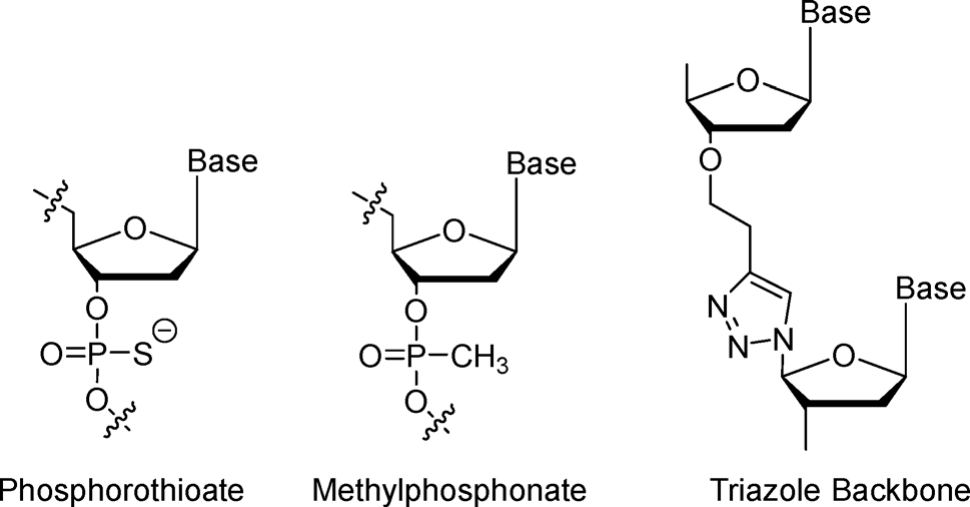
Structures of phosphorothioate, methylphosphonate and triazole backbone modifications.

Phosphodiester bond modifications are a major class of aptamer stability modifications that enhance nuclease resistance by altering the phosphate backbone (Fig. 5), typically through substitution of a non-bridging oxygen or by replacing the native linkage with a nuclease-orthogonal surrogate. By reducing recognition and cleavage by nucleases, these backbone changes improve biostability and can indirectly support longer functional persistence in biological matrices. Representative approaches include methylphosphonate and phosphorothioate substitutions, as well as full linkage replacements such as triazole backbones introduced *via* click chemistry, which may be implemented during selection or as post-SELEX optimization.^31^

#### 3.3.1. Methylphosphonate

Methylphosphonate (MP) modification replaces a non-bridging oxygen atom in the phosphate group with a methyl substituent, thereby altering the backbone chemistry in a way that can reduce nuclease recognition and cleavage.^68^ As a result, MP linkages are commonly used to enhance resistance to nuclease-mediated degradation, particularly against exonuclease-driven trimming. In practical systems, improved nuclease resistance is often reflected in increased persistence in serum; for example, MP-containing analogs—especially when combined with terminal inversion caps—have been reported to show markedly prolonged stability in human serum compared with unmodified counterparts.^69^ Mechanistically, the partial neutralization of backbone charge can also reduce local electrostatic repulsion and modulate duplex or secondary-structure stability, although the net structural effect is sequence- and context-dependent.

Moreover, MP modification is not universally stabilizing across all aptamer folds. Because it reduces the negative charge density of the phosphate backbone, MP substitution may destabilize certain conformations—particularly G-quadruplex architectures—where electrostatics and ion-mediated stabilization are critical.^68^ Therefore, MP linkages are often best positioned in regions where nuclease protection is needed but where perturbation of the functional fold is minimal, making them a useful option in post-selection optimization when balancing nuclease resistance against structural and binding constraints.

#### 3.3.2. Phosphorothioate

Phosphorothioate (PS) modification replaces a non-bridging oxygen atom in the phosphate backbone with sulfur, generating a P–S linkage that is substantially less susceptible to nuclease-catalyzed hydrolysis than the native phosphodiester bond. Accordingly, PS substitution is widely used as an aptamer stability modification to enhance resistance to nuclease-mediated degradation in serum and plasma.^70^ In many systems, improved nuclease resistance is reflected in greater persistence of intact aptamers in biological matrices and, as a downstream benefit, a longer apparent circulation half-life. For example, PS-containing designs have been reported to markedly slow degradation in plasma, and additional steric shielding (*e.g.*, *via* biotin–streptavidin architectures) can further reduce degradation rates.^71^

In therapeutic and preclinical constructs, PS substitution is often applied in a partial manner to balance stability gains with preservation of the binding-competent fold. For instance, introducing PS linkages at selected positions—such as in loop or nuclease-accessible regions of thrombin-binding aptamers—has been shown to maintain functional activity while improving resistance to DNase challenge and serum degradation. Related phosphorodithioate (PS2) variants, in which both non-bridging oxygens are replaced by sulfur, can further enhance nuclease resistance and, in some cases, alter folding in ways that strengthen binding through induced-fit effects.^72^ However, extensive PS/PS2 substitution is not universally tolerated: PS introduces chirality at phosphorus, producing diastereomeric mixtures, and high substitution levels have been associated with increased off-target interactions and toxicity in certain oligonucleotide contexts. These considerations often motivate a design preference for site-selective or partial PS substitution, used as part of a broader modification stack rather than as a blanket replacement.

#### 3.3.3. Triazole backbone *via* click chemistry

Triazole backbones, synthesized through click chemistry such as copper(i)-catalyzed azide–alkyne cycloaddition (CuAAC), fully replace phosphodiester linkages with 1,2,3-triazole units, creating an artificial scaffold unrecognizable to natural nucleases and thus highly resistant to degradation.^73^ This modification enhances chemical stability against hydrolysis and enzymatic cleavage, with triazole-linked DNA (TLDNA) or RNA (TLRNA) showing melting temperatures (*T*_m_) around 61 °C for certain analogs, and superior resistance to 5'/3'-exonucleases like DNase or snake venom phosphodiesterase.^74^ In aptamers, triazole integrations, often combined with locked nucleic acids (t-LNA), maintain duplex formation and enhance binding to RNA/DNA complements, as in G-quadruplex structures for thrombin targeting or hybrid triplexes. The neutral backbone reduces anionic charge, improving cellular uptake and biocompatibility, with examples in cyclic decoys stable in fetal calf serum and antisense oligonucleotides for gene modulation.^31^ However, changes in inter-base distances can disrupt precise stacking interactions required for high-affinity binding, often limiting applications to short, non-structured regions; for instance, internal triazoles may cause slight destabilization (Δ*T*_m_ ≈ −1.4 °C per modification) and sequence-dependent effects on hybridization. Copper-free variants mitigate toxicity, and templated CuAAC enables efficient ligation for aptamer cyclization, supporting therapeutic uses in CRISPR sgRNA or imaging probes.

### 3.4. Base modifications

Base modifications in aptamers involve introducing chemical groups to nucleotides, primarily to enhance resistance to nucleases by altering the molecular structure and interactions. The principle underlying these modifications is to increase the aptamer's structural diversity and hydrophobicity, thereby shielding the phosphodiester backbone from enzymatic cleavage while potentially improving binding affinity and pharmacokinetics.^31,60,75,76^

**Fig. 6:**
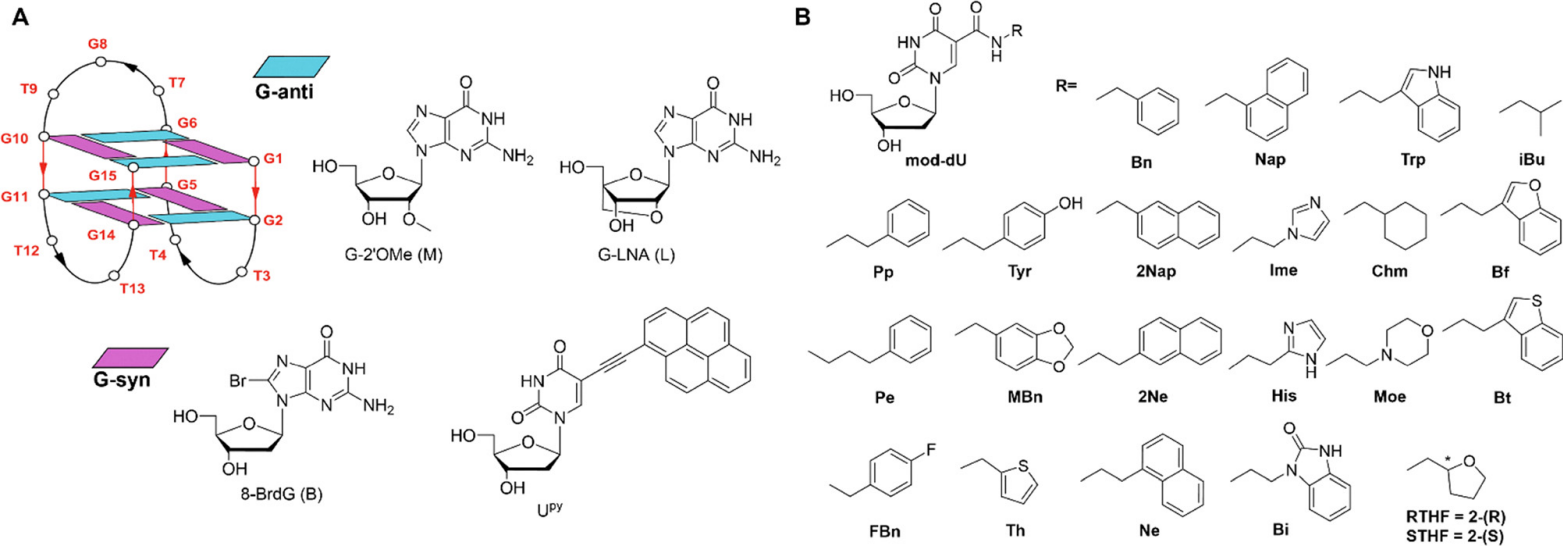
(A) Schematic representation of the TBA G-quadruplex structure and different base modifications; (B) 5′-modified uracil candidates in SOMAmers.

Thrombin binding aptamer (TBA, Fig. 6A) is one of the best-known G-quadruplex (G4)-forming aptamers that efficiently binds to thrombin, resulting in anticoagulant effects. Many researchers use chemical modifications on the bases to stabilize or strengthen the G4 structure.^77^ For 8' carbon-modified guanine, bromine substitution in TBA enhances anti-enzymatic properties by stabilizing the G-quadruplex structure, preventing unfolding and hydrolysis.^78^ Plavec *et al.* investigates the structural and functional effects of incorporating pyrene-modified uridine nucleotides (U^py^) into the TBA. The study systematically replaces thymine residues in the TBA loops with U^py^ to examine how the position of the modification influences G-quadruplex stability, structure, and biological activity, offering insights for designing functional aptamers with tailored properties for therapeutic and diagnostic applications.^79^

Slow off-rate modified aptamer (SOMAmer) technology represents a major breakthrough bridging nucleic acid and protein chemistry. For SOMAmers, uracil is modified at the 5' position with large hydrophobic side chains like benzyl, naphthyl, tryptophan, and isobutyl, forming a “hydrophobic core” akin to globular proteins (Fig. 6B). This core stabilizes the tertiary structure, spatially protecting the backbone and reducing degradation in biological fluids. This chemical expansion enables aptamers to form complex hydrophobic and π–π stacking interactions, resulting in extremely slow dissociation rates (binding half-lives ranging from hours to days) and affinity reaching picomolar or even femtomolar levels. SomaScan® platform (^1^) is a high-throughput proteomics technology based on over 11 000 unique SOMAmer probes, capable of detecting protein concentrations spanning 10 orders of magnitude in a single 55 µL sample.

### 3.5. Aptamer chirality inversion (spiegelmers)

Aptamer chirality inversion, exemplified by Spiegelmers, involves the use of l-nucleic acids to create mirror-image oligonucleotides that enhance biostability while preserving high-affinity binding to targets.^80^ This approach addresses the inherent susceptibility of natural d-nucleic acid aptamers to nuclease degradation in biological environments, making Spiegelmers particularly suitable for *in vivo* therapeutic applications across various disease targets.^31^

Spiegelmers are composed of l-nucleotides, which are the enantiomeric mirror images of natural d-nucleotides, resulting in inverted stereochemistry that forms left-handed helical structures. The development process employs a “mirror-image” SELEX (systematic evolution of ligands by exponential enrichment) strategy: a d-oligonucleotide library is selected against the enantiomeric (d-) form of the target, and the resulting aptamer sequence is then chemically synthesized in the l-configuration to bind the natural target.^81^ This chirality inversion confers exceptional resistance to nucleases, as these enzymes are stereospecific and cannot recognize or hydrolyze l-nucleic acids effectively. Stability studies demonstrate that Spiegelmers remain intact for over 60 hours in human serum at 37 °C, with half-lives exceeding 50 hours *in vivo*, far surpassing unmodified aptamers. Additional modifications, such as PEGylation (*e.g.*, attachment of polyethylene glycol), further extend pharmacokinetics by reducing renal clearance and improving bioavailability.

Despite the structural inversion, Spiegelmers retain high binding affinity and specificity, often in the picomolar to nanomolar range, through shape-based recognition and tertiary interactions.^82^ For instance, they exhibit reciprocal chiral specificity, binding D-configured targets with *K*_d_ values as low as 0.65 pM for VEGF-165 or 0.038 nM for IFN-γ, while showing minimal cross-reactivity with enantiomers.^83^ This affinity is maintained or enhanced post-optimization, enabling effective inhibition of target functions without triggering immune responses, as Spiegelmers display low immunogenicity in clinical studies.

*In vivo* applications leverage their biostability for therapeutic interventions, particularly in extracellular targeting of proteins, peptides, and small molecules. Spiegelmers have advanced to clinical trials, demonstrating safety in phase 1 studies with healthy volunteers and efficacy in phase 2 trials for various indications. Notable examples include NOX-A12 (olaptesed pegol), which targets CXCL12/SDF-1 to inhibit tumor progression in glioblastoma and chronic lymphocytic leukemia (CLL), improving survival and response rates (*e.g.*, 86% overall response when combined with chemotherapy) by mobilizing cancer cells and disrupting metastasis.^84^ NOX-E36 (emapticap pegol) antagonizes CCL2 to reduce inflammation in diabetic nephropathy, preventing glomerulosclerosis and improving glomerular filtration rate in preclinical models and phase 2a trials.^85^ NOX-H94 (lexaptepid pegol) targets hepcidin for anemia of chronic disease, increasing serum iron levels and preventing hemoglobin decline in animal models and clinical setting.^86^ Other applications encompass sepsis (NOX-D20 targeting C5a to attenuate inflammation and organ failure), endocrine disorders (NOX-1255 against GnRH), and viral detection (*e.g.*, distinguishing SARS-CoV-2 RNA motifs).^81,87^ These examples highlight Spiegelmers' versatility in addressing unmet needs in oncology, inflammation, and infectious diseases, with ongoing trials underscoring their translational potential.

### 3.6. Aptamer cyclization

Aptamer cyclization entails converting linear oligonucleotide sequences into cyclic forms, eliminating free 3′ and 5′ ends susceptible to exonuclease attacks. The principle is based on increasing structural rigidity and homogeneity, which enhances metabolic and thermal stability by preventing enzymatic access to termini, while the closed loop maintains or refines the aptamer's conformational integrity for target binding. However, excessive rigidity may reduce flexibility, potentially diminishing affinity if the active conformation is altered.^88^

Research interests has emphasized innovative cyclization techniques, such as CuAAC and photochemical locking, to create stable cyclic aptamers with minimal affinity loss.^89^ Advances include bioinspired template-directed ligation for single-round selection of circular aptamers, integrating L-RNA for mirror-image protection against nucleases.^90^ Studies also have combined cyclization with nanoparticle conjugation to improve delivery, addressing challenges like immunogenicity and pharmacokinetics *in vivo*.^91^ Circular bivalent aptamers have demonstrated improved thermal stability, with melting temperatures increased by more than 10 °C compared to double-stranded precursors, alongside enhanced tumor retention in mouse models.^92^ Notably, photochemically stabilized aptamers have improved conjugation efficiency, leading to better outcomes in mouse models for cancer therapy, paving the way for clinical translation.^93^

### 3.7. Multivalent aptamers

Multivalent aptamers are formed by linking multiple aptamer units—identical or heterogeneous—*via* spacers or scaffolds, leveraging avidity effects for enhanced performance. These molecules rely on cooperative binding, where multiple interactions increase overall affinity (often by 100-fold), while the larger size improves nuclease resistance, reduces clearance, and boosts cellular uptake through receptor clustering and endocytosis facilitation.^94,95^

The development of circular multivalent structures and DNA nanostructures are frequently reported for precise epitope targeting, enhancing stability in serum. Advances involve click chemistry for efficient assembly and hetero multivalent designs combining aptamers for dual targets like EGFR and PD-L1, improving immunotherapy synergy.^96^ Other studies have optimized spacer lengths to minimize steric hindrance, with research integrating nanomaterials for multimodal delivery.^97,98^

**Fig. 7:**
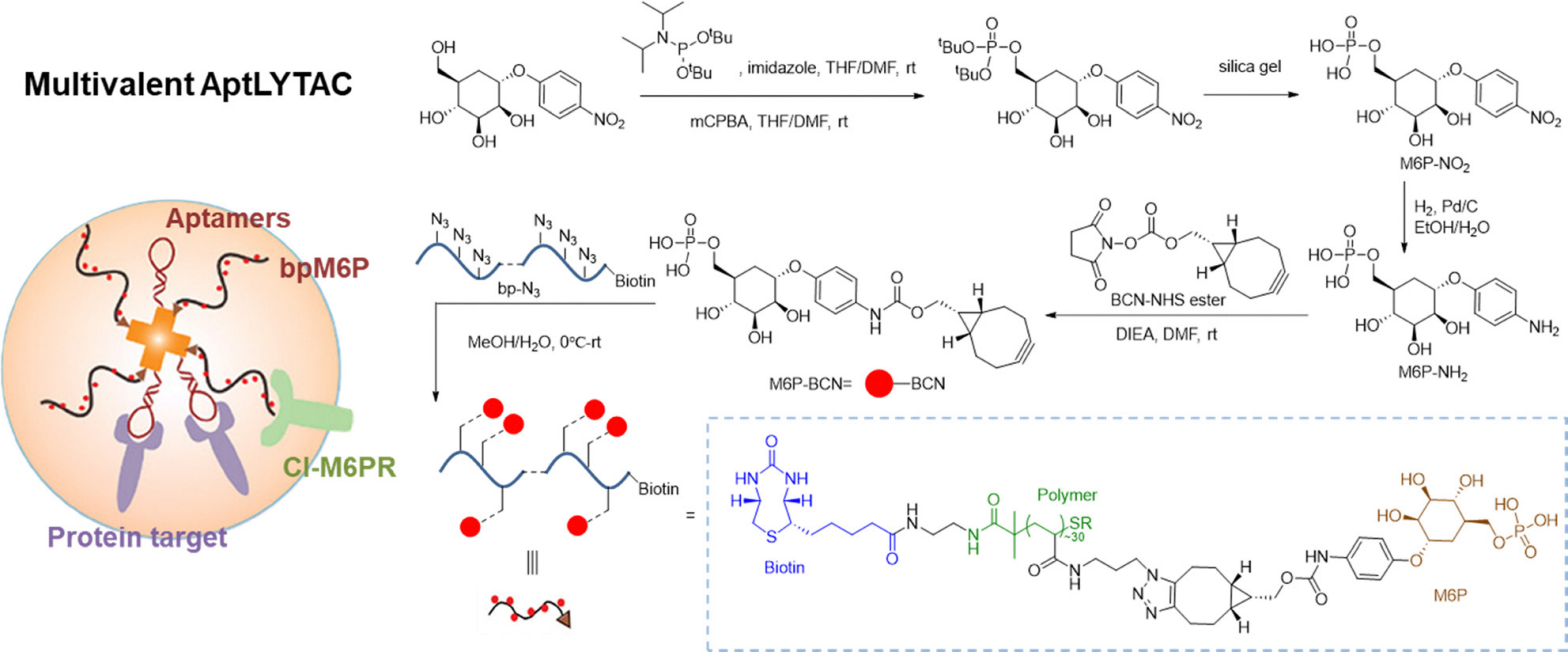
Multivalent aptamer-based lysosome targeting chimeras (LYTACs) platform for mono or dual targeted proteins degradation on cell surface.

Tan *et al.* reported a multivalent AptLYTAC platform, which uses biotin–streptavidin as a molecular scaffold for the degrader and employs the aptamer Sgc8 as a model, revealed that trivalent AptLYTACs achieve higher target protein degradation efficiency compared to monovalent designs (Fig. 7). Bastings *et al.* have developed the multivalent evolved DNA-based supramolecular assembly (MEDUSA) strategy. By directly introducing geometrically matched DNA scaffolds during the evolutionary process, this approach enables the synergistic screening of aptamers with multivalent binding capabilities.^99^ This not only yielded novel binding molecules with high specificity and protein interaction inhibitory activity but also facilitated the design of multivalent sensors capable of detecting target molecules through the modulation of scaffold rigidity and conformational switching. The strategy provides an integrated solution for the efficient targeting of oligomeric targets.

Multivalent aptamers, in contrast to bispecific antibodies (bsAbs), offer distinct advantages including rapid, low-cost chemical synthesis, superior tissue penetration owing to smaller molecular size (typically 20–100 kDa *versus* ∼150 kDa for bsAbs), and facile modular engineering for dual-targeting without requiring mammalian cell production. However, bsAbs benefit from established Fc-mediated pharmacokinetics and effector functions, whereas aptamer platforms still face critical bottlenecks: residual nuclease susceptibility despite multivalency, rapid renal clearance, and limited clinical translation data.^100^ These limitations can be mitigated through backbone modifications (*e.g.*, 2′-fluoro or LNA nucleotides), site-specific PEGylation or albumin-binding conjugates for half-life extension, and DNA nanostructure scaffolds that enhance serum stability while preserving cooperative avidity.^1^ Such hybrid strategies are expected to bridge the gap toward regulatory approval and synergistic immunotherapy applications.

While each modification class has been discussed individually above, their practical application requires understanding how they address distinct design bottlenecks. Table 2 provides a concise mapping of the major strategies onto key goals—exonuclease/endonuclease resistance, pharmacokinetic improvement, affinity preservation, and manufacturability—thereby serving as a practical decision-making framework for aptamer construct optimization.

**Table 2:** Mapping of aptamer stability modification strategies to key design goals

Modification strategy	Sub-type/example	Exonuclease resistance	Endonuclease resistance	PK improvement (half-life)	Affinity/specificity impact	Manufacturability/synthetic compatibility	Safety/immunogenicity notes	Representative clinical/preclinical rxamples
Terminal/end-capping	Biotin, 3′–3′ idT, BNA/LNA cap, eTNA	+ + + (steric block)	+	+ + (esp. with streptavidin)	Neutral/minimal	High (standard solid-phase)	Low	Macugen, many clinical candidates
End-conjugation	PEG (linear/branched), Lipid (cholesterol/DAG), GalNAc	+ + (hydrated shield)	+	+ + + (size ↑, lipoprotein association)	Neutral	Medium-high	PEG: anti-PEG Ab risk	Pegaptanib, Rondaptivon pegol, NOX-A12
Sugar ring	2′-F, 2′-OMe, 2′-NH_2_	++	+ + +	+	Variable (often preserved or ↑)	High (compatible with SELEX)	2′-F: occasional immune activation	Pegaptanib, Pegpleranib
Sugar ring	LNA, UNA, TNA, FANA, 4′-thio	++	+ + +	+	Often ↑ (rigidity or flexibility)	Medium (requires mutant polymerase for SELEX)	LNA: hepatotoxicity risk	Preclinical thrombin aptamers
Backbone	Phosphorothioate (PS/PS2), Methylphosphonate	+	+ + +	+	Variable (may ↓ if extensive)	High	PS: non-specific protein binding risk	ARC1779
Backbone	Triazole (click chemistry)	+ + + (orthogonal)	+ + +	+	May ↓ (backbone geometry change)	Low-medium (post-SELEX)	Generally low	Preclinical cyclic aptamers
Base modification	5-position hydrophobic (SOMAmer), 8-bromo-G	+	+ + (G4 stabilization)	+	Often ↑ (hydrophobic core)	Medium (special phosphoramidites)	Low	SOMAmer platform (SomaScan)
Chirality inversion	Spiegelmers (L-RNA/DNA)	+ + + (complete)	+ + + (complete)	+ + (with PEG)	Neutral (mirror-image SELEX)	Low (chemical synthesis only, no enzymatic amplification)	Very low immunogenicity	NOX-A12, NOX-E36, NOX-H94
Cyclization	Head-to-tail, photochemical lock	+ + + (no free ends)	++	+	Usually preserved	Medium (click or ligation)	Low	Preclinical circular bivalent aptamers
Multivalent assembly	Dimer/trimer, DNA nanostructure, AptLYTAC	+ + (size effect)	++	+ + + (avidity + size)	Often ↑ (avidity)	Medium (modular assembly)	Low	Trivalent AptLYTAC, MEDUSA

## 4. Discussion and perspective

As summarized in Table 2, robust *in vivo* performance is rarely achieved by a single modification but rather by rational stacking of complementary strategies that balance nuclease resistance with affinity and developability. In aptamer engineering, the pharmacodynamic effect is controlled by two interdependent but often competing parameters: nuclease resistance and target binding affinity. Although certain chemical modifications, including 2′-F and 2′-F arabinonucleotides (FANA), have been shown to simultaneously enhance stability and affinity, a reduction in affinity remains a common outcome of many aptamer stability modification strategies.^60^ Mechanistically, this reduction is associated with perturbations in ribose sugar ring conformation, alterations in backbone torsional flexibility, or steric/electrostatic interference at the binding interface—effects that may disrupt the precise structural complementarity required for high-affinity recognition.^1^ This trade-off was first observed in antisense oligonucleotides (ASOs), where phosphorothioate (PS) backbone modifications increased nuclease resistance but often reduced hybridization affinity.^101^ In aptamers, Ruckman *et al.* provided direct evidence that extensive 2′-O-methyl (2′-OMe) substitution can weaken molecular recognition without abolishing binding: comprehensive 2′-OMe modification of purines (excluding A5 and G20) in the VEGF-binding RNA aptamer t2.29 increased *K*_d_ by 3.5-fold, and t44.27 increased from 10 pM to 49 pM.^52^ These data confirm that there is a critical exists trade-off among stability, binding performance and developability in the design of therapeutic aptamers. Chemical modifications effectively enhance nuclease resistance and serum half-life, yet they frequently introduce structural perturbations that reduce target affinity or specificity. Conversely, unmodified aptamers suffer from poor biostability and rapid clearance, severely limiting their drug-like properties. Pre-SELEX strategies, incorporating modified nucleotides during library construction, offer a promising solution by evolving inherently stable and functional sequences, but they impose constraints on structural diversity, significantly increase screening complexity, and lower overall success rates. Thus, stability engineering should not aim for maximum protection globally but rather balance these factors through a rational hybrid approach—such as minimal post-SELEX modifications guided by structural insights or advanced DNA nanostructure scaffolds to ensure nuclease resistance while maintaining the folded structure of the therapeutic aptamer with excellent binding performance and developability.

The rapid development of machine learning models and other *in silico* tools has demonstrated great potential in predicting aptamer structures and simulating target-aptamer docking.^102,103^ However, there is currently no validated framework that can quantitatively predict how enzyme resistance stability modifications reshape binding thermodynamics and kinetics based on modification site, density, and combination patterns. As a result, stabilized aptamer design still depends heavily on empirical synthesis and screening, which slows translation and increases cost—especially when multiple chemistries are stacked.^104^ To accelerate modification site selection, practical near-term strategies include: (i) nuclease “hot-spot” mapping under relevant matrices to prioritize the earliest cleavage sites for protection; (ii) structure-aware, region-specific rules that protect termini and nuclease-exposed loops/junctions while modifying structural scaffolds conservatively; and (iii) a scan-then-stack workflow (small-window scans followed by selective stacking) to avoid combinatorial explosion. Systematic datasets generated from these workflows will also enable future models that explicitly learn modification–structure–binding relationships.

Regarding aptamer stability engineering and safety profiling, the assumption that nucleic acid therapeutics are inherently inert has been challenged by modification-associated adverse reactions in preclinical and clinical settings. For example, LNA-containing oligonucleotides have shown dose-dependent hepatotoxicity in some contexts.^105^ PEG conjugation can trigger anti-PEG antibodies and severe hypersensitivity; this risk contributed to termination of Pegnivacogin (a methoxy PEG-conjugated RNA aptamer targeting factor IXa) in phase II due to life-threatening allergic reactions.^106^ In parallel, dense modification stacks increase synthetic complexity and can compromise yield and reproducibility, weakening the manufacturing advantages of aptamers. Emerging mitigation strategies therefore emphasize using biocompatible carriers (*e.g.*, nanoparticles, liposomes) to provide steric shielding and persistence while maintaining the native aptamer fold, thereby reducing the need for heavily modified constructs.^107,108^ Overall, progress will likely come from combining minimal, hotspot-focused stability modification with data-guided site selection and risk-aware chemistry choices that account for safety and manufacturability constraints.

## Author contributions

Sifan Yu, Ge Zhang and Aiping Lu devised the project. Zhuoheng Pan, Yufei Pan and Huarui Zhang wrote the paper. All authors contributed to the article and approved the submitted version.

## Conflicts of interest

The authors declare no competing interests.

## Data Availability

No primary research results, software or code have been included and no new data were generated or analysed as part of this review.
